# Cognitive Impairment in Neuromyelitis Optica Spectrum Disorders: A Review of Clinical and Neuroradiological Features

**DOI:** 10.3389/fneur.2019.00608

**Published:** 2019-06-12

**Authors:** Frederike Cosima Oertel, Jana Schließeit, Alexander U. Brandt, Friedemann Paul

**Affiliations:** ^1^NeuroCure Clinical Research Center, Berlin Institute of Health, Charité – Universitätsmedizin Berlin, Corporate Member of Freie Universität Berlin, Humboldt-Universität zu Berlin, Berlin, Germany; ^2^Experimental and Clinical Research Center, Max-Delbrück-Centrum für Molekulare Medizin and Charité – Universitätsmedizin Berlin, Corporate Member of Freie Universität Berlin, Humboldt-Universität zu Berlin, and Berlin Institute of Health, Berlin, Germany; ^3^Faculty of Psychology and Neuroscience, Maastricht University, Maastricht, Netherlands; ^4^Department of Neurology, University of California, Irvine, Irvine, CA, United States; ^5^Department of Neurology, Berlin Institute of Health, Charité – Universitätsmedizin Berlin, Corporate Member of Freie Universität Berlin, Humboldt-Universität zu Berlin, Berlin, Germany

**Keywords:** neuromyelitis optica spectrum disorders, cognition, neuroinflammation, advanced imaging, MRI

## Abstract

Neuromyelitis optica spectrum disorders (NMOSD) are mostly relapsing autoimmune inflammatory disorders of the central nervous system (CNS) with optic neuritis, myelitis, and brainstem syndromes as clinical hallmarks. With a reported prevalence of up to 70%, cognitive impairment is frequent, but often unrecognized and an insufficiently treated burden of the disease. The most common cognitive dysfunctions are decline in attention and memory performance. Magnetic resonance imaging can be used to access structural correlates of neuropsychological disorders. Cognitive impairment is not only a highly underestimated symptom in patients with NMOSD, but potentially also a clinical correlate of attack-independent changes in NMOSD, which are currently under debate. This article reviews cognitive impairment in NMOSD and discusses associations between structural changes of the CNS and cognitive deficits.

## Introduction

Neuromyelitis optica spectrum disorders (NMOSD) are inflammatory autoimmune conditions of the central nervous system (CNS) with a typically relapsing course and a strong female preponderance (1–6). Key clinical features comprise optic neuritis, myelitis, and brainstem syndromes (7–13). Approximately 80% of the patients with NMOSD have pathogenic serum autoantibodies against aquaporin-4 (AQP4), a bidirectional water channel protein predominantly expressed by astrocytes, which is present all over the CNS (7, 14–21). AQP4 appears to not only be important for the internal water balance but its downstream mechanisms also seem to be essential for synaptic plasticity and neuronal functioning due to the involvement of astrocytes, although the exact mechanism of action is still unclear (22). In a subgroup of AQP4 antibody (AQP4-IgG) seronegative NMOSD patients as well as in patients with recurrent optic neuritis and a few patients with multiple sclerosis (MS) an antibody against myelin oligodendrocyte glycoprotein (MOG-IgG) can be detected (23–33). Nowadays, MOG-IgG seropositive patients are mostly assigned to a separate disease entity called MOG-IgG autoimmunity [or MOG encephalomyelitis (MOG-EM)], although they are formally still part of the NMO spectrum (5, 34–36).

In clinical routine, detection of AQP4-specific antibodies in serum allows for discriminating NMOSD from its most common differential diagnosis, MS. The high specificity of AQP4-IgG together with various immunological studies has made clear that NMOSD are not a variant of MS but a separate disease entity (7, 37–39). Subsequently, disease-modifying drugs used in MS, for example interferon-beta, glatiramer acetate, or natalizumab, were found to be ineffective or even harmful for patients with NMOSD (40–45). In contrast, current treatment strategies for patients with NMOSD comprise immunosuppressive therapies with azathioprine or mycophenolate-mofetil and B cell targeting therapies with rituximab (46–54). For most NMOSD patients, these drugs effectively reduce the attack frequency and attack-related accumulation of disability. However, recent studies suggested attack- and lesion-independent “covert” tissue damage in patients with NMOSD, from which clinical implications are not yet entirely clear (55–64) but which presumably contributes to attack-independent symptoms as of which one appears to be cognitive impairment (CI). CI as attack independent symptom was further supported by a study by Saji et al. (65) who tried to proof a permanent interaction between astrocytes and AQP4-IgG which lead finally to dysfunctional synaptic plasticity and hence could be involved in CI in NMOSD AQP4-IgG positive patients. Furthermore, even though CI appears to be a persistent and progressive symptom it still seems to be underrepresented in clinical monitoring and disability scores and often not sufficiently treated (66–70). Over the last years, those neuropsychiatric symptoms in NMOSD came to the forefront, and comparable larger cohorts in observational studies and the application of advanced imaging techniques allowed for the investigation of incidence and structural correlates of these neuropsychological symptoms. This article reviews CI in NMOSD and discusses associations between structural CNS changes and cognitive deficits.

## Cognitive Impairment in NMOSD

NMOSD show high rates of comorbidity with other physical and psychological conditions, including CI (67–69, 71). While cognitive dysfunctions have been commonly recognized as a burden in MS, this acknowledgment is still missing in NMOSD and hence studies investigating the link between NMOSD and CI are scarce (72, 73) despite NMOSD patients naming CI as one of their most relevant concerns (74) (see Table 1). In the few studies conducted, patients demonstrate a significant decrease of cognitive abilities and the prevalence of CI in different samples varies between 30 and 70% (71, 75–77). In addition to investigate cognition, each study has used its own assessment method to the end that one common disease specific cognitive test battery for patients with NMOSD is missing (71). Hence, meta-analyses such as the one of Meng et al. are difficult and are usually only able to analyse and make inferences from a small sample of studies (75). Currently, the most commonly used test battery appears to be the Rao's Brief Repeatable Battery of Neuropsychological tests (65, 76, 78). This battery assesses different aspects of cognition for example verbal memory, short and long term memory, processing speed, attention, concentration, and verbal fluency (65).

**Table 1:** The most important original publications on cognitive impairment in NMOSD.

**References**	**Country**	**Sample size**	**Portion of AQP4-IgG seropositive Patients**	**Neuro-psychological assessment**	**Main findings**
Blanc et al. (79)	France	30 NMOSD vs. 30 MS vs. 30 HCs	17/30	BRB-N CTT DST Go-no-go SDMT	-57% CI Compared to HC reduced scores in PASAT, SDMT, phonemic fluencies, the direct & indirect digit span test -compared to MS no differences
Blanc et al. (116)	France	28 NMOSD vs. 28 HCs	12/28	BRB-N CTT DST Go-no-go	-54% CI compared to HC decreased global & focal WM Correlation of WM volumes and cognitive test performance -no Gm differences
Cho et al. (118)	Korea	14 NMOSD vs. 21 HCs	13/14	BRB-N MACFIMS PASAT Digit Span Test DSCT BNT RCFT CVLT TMT COWAT SST	-patients perform significantly worse in attention/working memory, processing speed, executive function and visuospatial processing - CI in patients associated with local efficiency, regional efficiency and nodal clustering coefficient of two disrupted sub-networks
Fujimori et al. (78)	Japan	12 NMOSD vs. 14 MS	12/12	BRB-N WAIS-III WMS-R	-impairment in perceptual organization, working memory and processing speed -compared to MS less CI
He et al. (89)	China	22 NMOSD (acute relapse) vs. 22 patients with depression vs. 22 HCs	Not reported	CLOX CVLT II DST-backward PASAT SDMT	-compared to patients with depression reduced scores on immediate and short-term memory, information processing speed, and attention -suggesting that CI in NMOSD is not only due to depression but also due to cognitive connectivity distortions
Hyun et al. (85)	Korea	91 NMOSD vs. 52 MS vs. 44 HCs	Not reported	COWAT DST PASAT RCFT SDMT Stroop SVLT	-compared to HCs reduced thalamic volume in NMOSD and MS (more severe in MS), a finding mainly observed in Asian patient populations -association between CI and volume of the thalamus
Kim et al. (77)	Korea	82 NMOSD vs. 58 MS vs. 45 HCs	Not reported	COWAT Digit span test HVLT-R PASAT RCFT SDMT Stroop SVLT	-29 % CI -compared to MS less cognitive dysfunctions, especially in verbal learning, verbal and visual memory tests
Liu et al. (119)	China	26 NMOSD vs. 26 HCs	Only 18 patients tested: 16/18	/	-patients showed abnormal small-world network properties -regional efficiency of patients decreased in the visual, sensorimotor and default mode networks
Masuda et al. (115)	Japan	16 NMOSD vs. 20 MS	15/16	CAT TMT WAIS-III WMS-R	-compared to MS better performance in verbal memory, general memory and delayed recall and larger superior temporal gyrus volume -left superior temporal gyrus volume correlated with scores on the delayed recall
Moore et al. (71)	United Kingdom	42 NMOSD vs. 42 MS vs. 42 HCs	30/42	CVLT-II DKEFS DST Hayling phonemic and semantic SDMT	-67% CI substantial cognitive and psychiatric comorbidities in NMOSD -compared to MS greater psychological burden, but similar CI prevalence & profiles
Saji et al. (65)	Japan	14 NMO vs. 17 MS vs. 37 HCs	14/14	BRB-N	-57% CI compared to HC significant cortical neuron density decrease in layers II, III, and IV -cognitive deficits in sustained attention concentration, speed of information processing and verbal memory
Vanotti et al. (76)	Argentine	14 NMOSD vs. 14 MS vs. 14 HCs	Not reported	BRB-N SDMT Semantic fluency	-57% CI compared to HC dysfunctions in verbal fluencies & attention -compared to MS similar pattern of dysfunction

*BRB-N, brief repeatable battery of neuropsychological tests; BNT, Boston naming test; CAT, cognitive abilities test; CLOX, clock drawing executive test; COWAT, controlled oral word association test; COWAT, Controlled Oral Word Association Test; CTT, color trails test; CVLT, California verbal learning test; DGM, deep gray matter; DKEFS, Delis-Kaplan executive function system; DSCT, digit symbol coding test; DST, drive self-test; HCs, healthy controls; GM, gray matter; HVLT-R, Hopkins verbal learning test–revised; MACFIMS, minimal assessment of cognitive function in multiple sclerosis; MS, multiple sclerosis; NMOSD, neuromyelitis optica spectrum disorders; PASAT, paced auditory serial addition test; RCFT, Rey-Osterrieth complex figure test and recognition trial; SDMT, symbol digit modalities test; SST, spatial span test; SVLT, Shiraz verbal learning test; TMT, trail making test; WAIS, Wechsler adult intelligence scale; WCST, Wisconsin card sorting test; WM, white matter; WMS-R, Wechsler memory scale–revised*.

Studies investigating which aspects of cognition are most dysfunctional in patients with NMOSD depict ambiguous results: One of the first studies was conducted by Blanc et al. (79). They found alterations in attention, information processing and verbal fluency (79). The meta-analysis of Meng et al. concluded that patients with NMOSD perform generally worse than healthy controls and that patients are significantly worse in the areas of attention, language, memory, processing speed and executive function (75). Similar findings were made by Saji et al., who found that 57% of patients performed significantly worse in at least three cognitive tests compared to healthy controls (65). Furthermore, they found that deficits are predominantly overt in sustained attention, concentration, verbal memory, and speed of information processing (65). However, verbal fluency on semantic stimulation and spatial reasoning were intact (65). Opposed to these results, Vanotti et al. demonstrated a more pronounced dysfunction in the areas of attention and verbal fluencies (76). The variations across results are not only due to heterogeneity of samples including ethnic background, heterogeneity with regards to antibody status, and other interindividual differences, but also due to differences in assessment, the definition and percentage of CI in samples, correction for depression, as well as analysis of magnetic resonance imaging (MRI) and overall differences in study design (72). In particular, the heterogeneous antibody status and MOG-IgG could be a major confounder, as many studies had mixed samples for example Blanc et al. (17 AQP4-IgG seropositive patients/13 AQP4-IgG seronegative patients who were not tested for MOG-IgG) (79). Other studies for example Vanotti et al. or Liu et al. have not reported antibody status, and hence may have missed a possible association between antibody status and CI (76, 80). Further constraints entail that most of the studies recruited rather small sample sizes, ranging from 12 to 91 patients, and cover only a limited spectrum of ethnic backgrounds, with most studies being from Asian countries (71). Thus, while CI seems to be commonly present in a high percentage of NMOSD patients the specific domain of dysfunctional performance varies greatly between studies and samples. It appears that the most common cognitive dysfunction across all studies is decline in attention and memory performance. Moreover, the question as to the whether NMOSD pathobiology is causative for CI has not been clarified.

## AQP4-IgG and Cognitive Impairment

Currently, attack-independent structural changes as part of the pathology in NMOSD are a matter of debate (56–59, 64, 81, 82). The continuous and sometimes deteriorating neuropsychological symptoms might point toward a smoldering disease process in NMOSD independent of clinical attacks, for instance directly caused by the pathogenic antibody AQP4-IgG (56–59). Hence, few studies have tried to investigate the interplay of AQP4-IgG, which usually are a persistent disease factor, and cognitive impairment. Fan et al. investigated the link between AQP4-IgG and cognitive functioning, in particular spatial memory, in mice: They concluded that AQP4-IgG appear to inhibit neuronal plasticity and thus lead to a worsening in memory consolidation and spatial memory, as mice AQP4 knockout mice showed deficits in the acquisition and reversal phase in the Morris water maze (83, 84). This link is further supported by Skucas et al. (86) who found impaired long term potentiation and thus deficits in spatial memory in AQP4 knockout mice (83). While this finding would explain deficits in spatial memory, it provides no explanation for attention deficits, which are often observed in NMOSD patients. An attempt to explain the full extent of poor cognitive functioning in patients with NMOSD was made by Saji et al. who claimed that a unique dynamic between AQP4-IgG and astrocytes is the underlying mechanism belonging to CI (65). According to Saji et al. the unique dynamic exhibited by AQP4-IgG is leading to substantial diffuse cortical neuronal loss throughout the whole brain and hence may lead to neurodegeneration independent of clinical attacks (65). Thus, in contrast to MS where the disease causes demyelinating lesions, AQP4-IgG seropositive NMOSD results in pathological changes of the gray matter especially in the cortical layer II (65). These processes are suggested to include a disruption of glutamate and/or water homeostasis and thus excitotoxicity, release of soluble neurotoxic factors, which may trigger neurodegeneration by diffusing into the gray matter (GM) (65). This pathomechanism would further explain the GM atrophy observed by some studies in patients with AQP4-IgG seropositive NMOSD (80, 85). Other processes possibly induced by downstream mechanisms of AQP4 dysfunction are the activation of the innate immune system and hence activation of microglia as well as other autoimmune properties that could be involved not only in AQP4-IgG seropositive NMOSD but also as response to AQP4-IgG (37, 65, 86, 87). The hypothesis of an immune reaction, leading to pathological downstream mechanisms is further supported by Takeshita et al. (38). They found that, in cell cultures, AQP4-positive astrocytes produced interleukin-6 when exposed to AQP4-IgG. The cytokine interleukin-6 was found to disrupt endothelial cell functioning, which results in impaired blood barrier function (38, 88). On top of that, AQP4 expression seems to play an important role in regulating synaptic plasticity, which might be altered in NMOSD due to AQP4-IgG (88). Although the exact mechanism remains unclear, the existence of CI and other neuropsychological symptoms in AQP4-IgG seropositive NMOSD points toward an attack-independent pathology, potentially induced by the pathogenic antibodies themselves.

## Link Between Cognitive Impairment and Depression in NMOSD

Depression is known to also be a common and insufficiently treated symptom in NMOSD: Whereas around 50% of moderately to severely depressed patients with NMOSD receive antidepressant medical treatment, only 50% of treated patients report satisfactory treatment responses (68). Nevertheless, only few studies have investigated the link between depression and CI in patients with NMOSD. On the one hand, studies found a strong association between poor cognitive performance and high levels of depressive symptoms (71, 76). On the other, the study of Blanc et al. reported no association between cognition and depression (79). These opposing results could be explained by small sample sizes, heterogeneous cohorts, and different ethnic backgrounds. Furthermore, as both CI and depression tend to have high prevalence in NMOSD, an association but not causation could be possible (67, 71). On top of that, even if a causal link could be proven, the direction of this association would still be questionable. Especially, since studies focusing on fatigue and quality of life are implying a role of these in depression as well as in cognition (89–91). Therefore, it is advisable to investigate the full spectrum of the disease and its psychological comorbidities instead of only exploring the link between depression and CI and thus eliminating possible confounders.

## Association Between Cognitive Impairment and Structural Changes

Numerous studies have described brain tissue alterations in MS [global atrophy, microstructural damage of normal-appearing white matter (WM) and GM)], but studies investigating MRI characteristics in NMOSD are still scarce (92–98). According to the current state of knowledge, up to 80% of AQP4-IgG seropositive NMOSD patients present with cerebral lesions in AQP4-rich sites for example the hypothalamus and periependymal regions and—in contrast to MS—cortical lesions are usually absent (99–105). Also, the few existing MRI studies on MOG-IgG seropositive encephalomyelitis suggest a high similarity with MRI features of AQP4-IgG positive patients with the occasional incidence of characteristic fluffy brainstem lesions (106–108). However, several groups recently reported seizures with cortical MRI involvement on MRI in MOG-IgG seropositive patients which is considered very rare in AQP4-IgG positive NMOSD (109, 110). Whereas, several studies exist describing transsynaptic damage after optic neuritis and myelitis, the existence of global atrophy, and diffuse tissue alterations of WM and GM in AQP4-IgG seropositive NMOSD is still a matter of debate (13, 58, 63, 64, 93, 95, 111, 113, 114).

In order to explain the cognitive dysfunction observed, studies have focused on cortical abnormalities (80, 115). While in MS cognitive dysfunction is linked to cortical lesions, no such correlation can be observed in patients with NMOSD (71, 79, 80, 115). In the study of Liu et al. (80) when comparing 54 NMOSD patients, 28 of which were cognitively preserved and 26 of which had CI, there was no association found between overall brain lesion load and CI (76, 80). Some studies found a correlation between WM and GM atrophy, and CI: Blanc et al. linked focal WM volumes of the brainstem, cerebellum, corticospinal tract, corpus callosum, longitudinal fascicle, and inferior longitudinal fascicle with general CI in NMOSD patients (79). In particular, visual memory, verbal memory, speed of information processing, short term memory and executive functions were found to be impaired (116). Hence, both focal and global WM volume were linked to CI, but no GM atrophy was observed (116). This finding is in line with the work of Finke et al., who observed no changes in deep GM volumes in AQP4-IgG seropositive NMOSD (112). This is further underlined by so far unpublished results from our groups suggesting missing atrophy of the entire thalamic volumes in AQP4-IgG seropositive patients compared to HCs and only selective subnuclei atrophy in attack-related nuclei such as the lateral geniculate nucleus (117). Conflicting with the missing GM and thalamus atrophy was the study conducted by Hyun et al. (85). They described a significant link between volume of the thalamus and CI in patients with NMOSD, with reduced volumes in patients with poorer cognitive performance (85). Nevertheless, the different conclusions about thalamus volume could be due to the fact that Hyun et al. (85) measured their patients after a mean disease duration of 8 years where advanced degeneration has taken place, which potentially could not only be a confounder on its own but also could possibly lead to confounding through depression, advanced disability, and pain. Furthermore, the sample population appears to play an important role when comparing results in respect to GM atrophy, as reduced volume is mainly observed in Asian samples while studies examining a Caucasian sample fail to replicate these results (117). Hence, studies examining cortical volumes in NMOSD should be interpreted carefully.

Further studies investigating structural changes indicate that white matter network alternations could be the underlying reason for cognitive decline in some NMOSD patients (118, 119). One study by Liu et al. investigated the structural connectivity of 26 NMO-patients and 26 sex- and age-matched HCs with help of diffusion tensor tractography (DTI) (119). After performing network analysis, they found alternations in the small-world topology of the white matter structural networks, including abnormal parameters in path length, an increase in small-worldness as well as an increase in normalized clustering. Furthermore, they found an altered global network organization, which is in line with reduced cognitive efficacy observed in NMOSD patients. They suggest that in particular the reduced efficacy of the precuneus (PCUN), a hub belonging to the default-mode network, which is highly involved in cognitive processing, could partially contribute to CI in patients. A similar DTI study investigating white matter networks and cognitive dysfunction in NMOSD was performed by Cho et al. (118). They enrolled 14 NMOSD patients and 21 HCs, and could confirm the finding of Liu et al., that global network strength is decreased (118, 119). Furthermore, they indicated two disrupted sub-networks, each consistent of six hub nodes, including the PCUN. The disrupted networks were significantly linked to poor performance in attention, processing speed and working memory, as well as to visuospatial processing and executive functions. In particular, the local efficiency, regional efficiency and clustering coefficient of these two sub-networks appear to play a role in CI in NMOSD.

Hence, while several structural changes in GM as well as in WM networks seem to occur in NMOSD it appears to be rather difficult to link CI with one particular tissue alternation. On top of that these studies that have investigated the underlying structures of CI face limitations of which a mixed cohort with heterogeneous antibody-status of the patients is one of the most prominent (96).These limitations, in particular the heterogeneous antibody-status, with earlier studies like Blanc et al. (79, 116) reporting a higher seronegative-seropositive ratio than current studies, hamper comparison between studies.

## Outlook

Cognitive impairment (CI) appears to be one of the more prominent progressive and attack independent symptoms of NMOSD. Hence, a sensitization of the treating neurologists as well as early and standardized screening tests are therefore necessary to improve the management and treatment of cognitive impairment and other neuropsychiatric symptoms in NMOSD. In the future, adequately powered studies investigating CI, its underlying pathobiological mechanisms as well as longitudinal changes and clinical impact should be a priority of NMOSD research.

With regards to NMOSD-specific pathology, continuous and sometimes deteriorating neuropsychological symptoms might point to covert disease activity in NMOSD independent of clinical attacks. In light of the ongoing discussion on attack-independent structural changes in NMOSD, we should therefore keep in mind that CI might represent a clinical correlate of underlying subtle microstructural CNS changes.

## Author Contributions

FO, JS, AB, and FP wrote the manuscript as well as read and approved the final version.

### Conflict of Interest Statement

FO was employed of Nocturne, unrelated to this manuscript. AB is founder and holds shares of Motognosis and Nocturne. He is named as inventor on several patent applications describing serum biomarkers for MS, perceptive visual computing for tracking of motor dysfunction and OCT image analysis. FP reports research grants and speaker honoraria from Bayer, Teva, Genzyme, Merck, Novartis, MedImmune and is member of the steering committee of the OCTIMS study (Novartis), all unrelated to this work. The remaining author declares that the research was conducted in the absence of any commercial or financial relationships that could be construed as a potential conflict of interest.
